# Associations of estrogen with modifiable and non‐modifiable risk factors for dementia: A narrative review

**DOI:** 10.1002/alz.70873

**Published:** 2025-11-20

**Authors:** Sarah Gregory, Katie Bridgeman, Hannah Darwin, Mariapaola Barbato, Laura Booi, Anna Brugulat Serrat, Antonella Santuccione Chadha, Francesca R. Farina, Natalie Jenkins, Otto‐Emil I. Jutila, Audrey Low, Roberta Marongiu, Lorina Naci, Polly Pulford, Craig W. Ritchie, Karen Ritchie, Chinedu Udeh‐Momoh, Tamlyn Watermeyer, Miles Welstead, Graciela Muniz‐Terrera

**Affiliations:** ^1^ Edinburgh Dementia Prevention Centre for Clinical Brain Sciences Outpatients Department 2 University of Edinburgh Western General Hospital Edinburgh UK; ^2^ Scottish Brain Sciences Edinburgh UK; ^3^ School of Medicine University of St. Andrews St. Andrews UK; ^4^ Women's Brain Foundation Basel Switzerland; ^5^ Global Brain Health Institute Trinty College Dublin Dublin Ireland; ^6^ Centre for Dementia Research Leeds Beckett University Leeds UK; ^7^ Barcelonaβeta Brain Research Center (BBRC) Pasqual Maragall Foundation Barcelona Spain; ^8^ Centro de Investigación Biomédica en Red de Fragilidad y Envejecimiento Saludable (CIBER‐FES) Instituto de Salud Carlos III Madrid Spain; ^9^ Global Brain Health Institute University of California San Francisco San Francisco California USA; ^10^ Pritzker School of Medicine University of Chicago Chicago Illinois USA; ^11^ School of Psychology and Neuroscience University of Glasgow Glasgow UK; ^12^ Department of Psychology The University of Edinburgh Edinburgh UK; ^13^ Department of Psychiatry School of Clinical Medicine University of Cambridge Cambridge UK; ^14^ Department of Neurosurgery Weill Cornell Medicine New York USA; ^15^ Trinity College Institute of Neuroscience School of Psychology Trinity College Dublin Dublin Ireland; ^16^ South West London & St George's NHS Trust Springfield University Hospital London UK; ^17^ Institut de Neurosciences de Montpelier (INM) Inserm Montpellier France; ^18^ Institut du Cerveau Trocadéro Paris France; ^19^ Division of Clinical Geriatrics Department of Neurobiology, Care Sciences, and Society Karolinska Institutet Stockholm Sweden; ^20^ School of Medicine Wake Forest University Winston‐Salem North Carolina USA; ^21^ Brain and Mind Institute Aga Khan University Nairobi Kenya; ^22^ Ellison Place Northumbria University Newcastle upon Tyne UK; ^23^ Ohio University Heritage College of Osteopathic Medicine Ohio University Athens Ohio USA

**Keywords:** contraception, dementia, estrogen, hormone therapy, menarche, menopause, pregnancy, women's health

## Abstract

Female sex is associated with higher incidence and risk of dementia. Estrogen may represent one important mechanism contributing to the increase in incidence rates. In this review, we synthesize narratively the evidence for associations between estrogen—across the life course from menarche to menopause, estrogen‐containing hormonal contraception and hormone replacement therapies, and pregnancy—with potential modifiable risk factors for dementia. These include education, hearing loss, traumatic brain injury, hypertension, alcohol use, obesity, smoking, depression, physical inactivity, diabetes, low‐density lipoprotein (LDL) cholesterol, social isolation, air pollution, and untreated visual loss, as well as apolipoprotein E ε4. In addition, evidence is summarized for associations with sleep, diet, and stress. Evidence suggests that estrogen is associated with some of these modifiable risk factors for dementia, particularly LDL cholesterol, smoking, and depression. Research needs to further define these associations and understand whether interventions targeting estrogen levels at key life stages could offer intervention opportunities to reduce future risk of dementia in women.

**Highlights:**

- Higher dementia risk in women may be associated with estrogen.
- Estrogen is associated with some of the modifiable risk factors for dementia.
- However, significant gaps exist in the literature for most risk factors.

## 1 BACKGROUND

Female sex is increasingly recognized as a major risk factor for Alzheimer's disease (AD) and related dementias (ADRDs),^1^ with higher incidence and risk observed in women.^2^ Sex hormones may contribute to sex differences in AD pathogenesis and progression.^3^ In particular, declining sex steroid concentrations (testosterone,^4^ estrogen^5^) may be an important risk factor in deteriorating brain health. Menopause, characterized by a sharp decline in estrogen levels, may offer a critical window for intervention. Notably, menopause has been identified as a priority in the recent Women's Health Strategy for England.^6^ Later age at menarche, which can reflect a shorter lifetime exposure to endogenous estrogen, has also been associated with an increased risk for dementia in multiple systematic reviews.^7^ In contrast, later menopause was found associated with a lower risk of dementia.^7^ Recent studies suggest that apolipoprotein E (*APOE*) ε4 confers greater AD risk in females than males via interaction with estrogen levels.^8^, ^9^

Endogenous estrogen exists in three forms, which fluctuate throughout the lifespan: estrone (E1), estradiol (E2), and estriol (E3;^10^ see Table 1). Estrone is the only naturally produced form of estrogen that continues to be produced after menopause. Estradiol is the predominant circulating estrogen during reproductive and ceases to be produced during menopause. Estradiol exerts a wide range of effects on neuronal structure and function, including maintaining metabolic homeostasis, and regulating glucose metabolism, oxidate phosphorylation/stress, and neuronal ATP generation.^11^, ^12^, ^13^ In most studies, references to “estrogen” typically imply estradiol. Synthetic forms of estradiol are the most common form of estrogen used in hormone replacement therapy (HRT) and some oral hormonal contraceptives,^14^ including ethinylestradiol (synthetic estradiol), estetrol (E4, a naturally occurring estrogenic steroid hormone), and mestranol (another synthetic estradiol). Estriol is the third type of estrogen and increases specifically during pregnancy.^15^ Estrogens are synthesized primarily in the ovaries but can also be synthesized in the adrenal glands and adipose tissue,^16^ and in the brain.^17^ The synthesis pathway begins with the enzymatic conversion of cholesterol to pregnenolone, and ends with the conversion of testosterone to estradiol.^18^

**TABLE 1:** Overview of estrogens, both naturally occurring and synthetic.

Estrogen type	Natural/synthetic	Period of life course
Estrone (E1)	Naturally occurring	Primary form of estrogen present after menopause.
Estradiol (E2)	Naturally occurring	Most abundant form of estrogen. Regulates menstrual cycle and supports fertility. Significant decline in levels during menopause.
Estriol (E3)	Naturally occurring	Supports pregnancy and fetal development.
Ethinyl estradiol	Synthetic	Used in contraception, HRT, medications to manage menstrual disorders, and prostate cancer.
Estetrol (E4)	Natural estrogenic steroid hormone Naturally occurring	Contraception. Produced by the fetus during pregnancy.
Mestranol	Synthetic estradiol	Used in contraception, HRT, and medications to manage menstrual disorders. Precursor of ethinyl estradiol

Abbreviation: HRT, hormone replacement therapy.

In terms of exogenous estrogen, combined oral contraceptives are used by an estimated 5% to 20% of women aged 15 to 49 years worldwide,^19^, ^20^ and HRT in women aged 45 to 69 years in Europe ranges from 5% to 25% of the population,^21^ whereas recent data from the United States suggests a decline from 26.9% in 1999 to just 4.7% in 2020.^22^ In addition, estrogen is the main therapy for transgender women, and the global demand for gender‐affirming hormone therapy is increasing.^23^, ^24^ The use of oral contraceptives, which reduce endogenous steroid hormone levels, has been linked to changes in functional connectivity typically associated with high levels of progesterone, characterized by increased prefrontal and decreased parietal connectivity.^25^ There is inconsistent evidence for the association between HRT and dementia, likely influenced by factors such as age, genetic background, and timing of use.^26^, ^27^, ^28^, ^29^, ^30^ The only randomized trial to date investigating the effects of HRT on dementia incidence found a doubling of the risk of all‐cause dementia with estrogen‐plus‐progestogen therapy.^31^ Two recent randomized trials in younger postmenopausal women (closer to menopause transition) found neither harm nor benefit of HRT on cognition.^32^ Understanding more about the timing of HRT intervention relative to menopause is important to contextualize these apparently conflicting findings.

In this work, we narratively synthesize the evidence of whether/how estrogen (1) relates to established modifiable and non‐modifiable risk factors of dementia (for factors as detailed in the latest Lancet Commission on dementia prevention, intervention, and care^33^); (2) confers protective effects of its exogenous form, for example, HRT; and (3) moderates the adverse effects of risk factors on brain health and cognitive impairment. In addition to the 14 identified modifiable risk factors for dementia included in the Lancet Commission, we reviewed evidence for the potential risk factors of sleep, diet, infections, and stress. All these risk factors are highlighted by the Lancet Commission group as potentially modifiable but currently lacking a consistent evidence base. However, all are likely to be important to consider through the lens of sex differences and therefore are included here. The initial literature identification and synthesis was performed by three authors (S.G., K.B., H.D.). We searched PubMed and Google Scholar for articles published from database inception to December 1, 2024, that examined the association between modifiable risk factors or *APOE* ε4 and estrogen, hormonal contraception, HRT, pregnancy, or menopause. A list of Medical Subject Heading (MeSH) and free‐text search terms is included in the Appendix. Identified articles were reviewed by S.G., K.B., or H.D. with consensus meetings among the three to determine relevance for inclusion in the study. Additional authors added key papers relevant to their area of expertise and S.G. reviewed all additions before circulating the final draft. Studies were included in a narrative synthesis process, selected due to the breadth of risk factors included. Use of terminology has been driven by the included articles, with either biological sex (e.g., female, male) or gendered terms (e.g., woman, man) reported as appropriate. All authors reviewed the draft and added additional comments before submission.

## 2 ASSOCIATIONS BETWEEN EARLY‐LIFE MODIFIABLE RISK FACTORS FOR DEMENTIA AND ESTROGEN

### 2.1 Education

In an analysis of female workers who were part of the Korea National Health and Nutrition Examination Survey, fewer years of education were associated with more irregular menstrual cycles compared to more years of education.^34^ Other important factors that also impacted cycle irregularity included older age at menarche, childbirth, obesity, and shift work. This suggests an indirect association between education and menstrual cycle regularity, possibly mediated by obesity, or other unmeasured variables. Studies have investigated associations between education attainment and age at menopause; however, there is limited evidence to suggest a significant relationship between these factors. Some, but not unequivocal, evidence has been found for an association between higher education and later age at natural menopause.^35^, ^36^ In a study of community‐dwelling older women in Greece, an association between increased education levels and better cognitive performance in later life was found, which was stronger in women who experienced earlier menarche (ages 11–14 years) compared to those with later menarche (ages 15–17 years), for whom positive associations were limited to secondary education, with no benefit from tertiary education.^37^ These findings point to a possible critical time window during which education may be particularly beneficial through interactions with the neuroprotective and neurodevelopmental effects of estrogen. However, the possible impact of confounding factors such as socioeconomic status cannot be discounted. Higher levels of education were associated with better cognitive performance in postmenopausal—but not perimenopausal—women in a small cross‐sectional study of Finnish women,^38^ suggesting that the cognitive benefits of education may be more evident when estrogen levels are most depleted. Larger studies are needed to replicate this finding before firm conclusions can be drawn. During pregnancy, higher education has been associated with lower estrone and estradiol levels at 10 to 12 weeks’ gestation in Hispanic and Black women but not in White women.^39^ No other studies were identified that reported on associations between education and estrogens during pregnancy, highlighting a gap in literature.

Regarding HRT, there have been reported differences in access between women with no formal qualifications and those with at least one qualification, with the former more likely to seek advice on menopause symptoms and be recommended HRT by their primary care practitioner.^40^ The authors suggest this may be due to patients with at least one qualification being more successful at sourcing information about menopause and HRT, and more likely to ask questions about risks of treatment. This aligns with a strong evidence base that demonstrates health literacy to be associated with a broad range of health outcomes.^41^ Lower education attainment (below college level) has also been associated with a higher likelihood of experiencing vasomotor symptoms (e.g., hot flashes, cold sweats, night sweats) during menopause.^42^, ^43^, ^44^ Several explanations have been proposed, including mediating factors such as obesity and differences in health‐seeking behaviors that may lead to better management of symptoms. Quality of life during perimenopause has also been linked to education, with more years of education associated with better self‐reported quality of life.^40^, ^42^ While the mechanisms behind this are unclear, they are likely to be complex and multifactorial, including key socioeconomic, lifestyle, and health behavior factors.

There remains limited evidence on associations between education and estrogen across the life course, with more research needed, especially outside of European countries, to understand any important associations with this modifiable risk factor for ADRD.

## 3 ASSOCIATIONS BETWEEN MIDLIFE MODIFIABLE RISK FACTORS FOR DEMENTIA AND ESTROGEN

### 3.1 Hearing loss

Hearing loss has been associated with changes in estrogen across the life course in various studies. A rare occurrence during pregnancy is sudden deafness, which may in part be driven by a significant increase in estrogen causing thromboembolic episodes of the cochlear artery or disruptions to inner ear homeostasis.^45^ No studies were identified that looked at broader associations between hearing and estrogen during pregnancy. Previous research has shown that a longer exposure to estrogen is associated with better preserved hearing.^46^, ^47^ Decreases in estrogen levels lead to a negative impact on hearing due to changes in blood flow level in the cochlear nucleus.^48^ Untangling what the impact of estrogen is, independent of the aging process, will be important to uncover in future work. Hearing loss may also be indirectly related as a marker of more general brain aging cortical loss, particularly in frontal areas.^49^, ^50^

Differences in auditory processing and hearing performance have been found between perimenopausal and postmenopausal women, with those who have been through menopause exhibiting more hearing difficulties.^51^ Older age at menopause, a longer lifetime exposure to estrogen, and use of HRT were found to result in better hearing in a study of postmenopausal Korean women (*n* = 3653).^46^ Those with longer estrogen exposure had superior hearing on a range of frequencies on pure‐tone audiometry. Hearing loss was reduced in those taking HRT, particularly for high‐frequency hearing levels. An analysis of data in the Nurses’ Health Study II conversely found that older age at natural menopause was associated with a higher risk of hearing loss, with HRT, and particular longer duration of use, associated with higher risk.^52^ Findings related to hearing loss and HRT may differ depending on the treatment used, with combined estrogen/progesterone therapy associated with negative impacts on hearing compared to estrogen monotherapy (*n* = 30).^53^ Estrogen supplementation has been found to delay hearing loss in postmenopausal women, with 17β‐estradiol HRT users experiencing better hearing performance compared to non‐HRT and combined HRT groups.^54^ The protective effects of estradiol may be most significant for women aged 40 to 60 years, with higher estradiol levels associated with less speech‐frequency hearing loss in US women.^55^ Women who underwent surgical rather than natural menopause (*n* = 20) have also been found to experience more severe hearing decline,^56^ potentially due to the abrupt estrogen loss.

Overall, there is evidence to suggest that estrogen may be linked with hearing loss, with exposure to estrogen for longer periods of a woman's life associated with a reduced risk for hearing loss. However, there remains some inconsistency in the evidence, and understanding the role of estrogen alongside, and independent to, the aging process will be important for future research efforts. Some of the studies in this area have included comparatively small sample sizes, and as such must be interpreted with caution, with a need for larger studies to provide more robust evidence.

### 3.2 Traumatic brain injury

Traumatic brain injury (TBI) is a known risk factor for the development of ADRD.^33^, ^57^, ^58^ Studies in rodents suggest there may be a neuroprotective role for estrogen that contributes to different outcomes post‐TBI for males (worse outcomes) and females^59^; however human studies remain limited.^60^, ^61^, ^62^ Estrogen is thought to influence neuron recovery after TBI and promote neurogenesis, and when given after an injury in pre‐clinical studies, estrogens have been found to reduce pro‐inflammatory and increase anti‐inflammatory cytokines, reducing damage caused to the brain.^63^ Estradiol can also enhance cerebral blood flow thus reducing vascular and blood–brain barrier damage after a TBI.^60^

However, evidence of a neuroprotective effect of estrogen in human studies is not clearly understood. Supporting the hypothesis that estrogen has a neuroprotective effect, younger women of reproductive age, with the highest levels of circulating estrogen, have been shown to experience reduced secondary injury and improved outcomes after TBI compared to postmenopausal women.^59^ Studies have also shown that the neuroprotective actions of estrogen and progesterone decline with advancing age, potentially exacerbating poorer outcomes in older women post menopause.^62^, ^64^ Conversely, however, some studies report better outcomes in postmenopausal women.^62^, ^65^ Davis et al.^62^ report better mortality outcomes in postmenopausal women but not premenopausal women compared to men. In addition to mortality, it has also been shown that from menarche to menopause, women have higher post‐concussion scores compared to men.^66^ These findings suggest that estrogen is not neuroprotective and may be associated with worse outcomes.

Interestingly, when a woman has experienced a concussion and is using oral contraceptives containing estrogen and progesterone, there is evidence of better recovery from concussion, including reduced severity and number of post‐concussion symptoms.^67^ Hormonal contraceptives generally lower the levels of circulating hormones, although, while this supports the notion that estrogen and progesterone are not neuroprotective, it may potentially point to a mechanism of protection against a drop in circulating gonadal hormones which occurs after a head injury.^67^ There remains significant controversy in the literature regarding the effect of estrogen on TBI outcomes.

To date, most of the research exploring the relationship of the effect of estrogen on TBI recovery has been conducted in a laboratory setting (mainly rodent studies) and more clinical research is required to fully understand this relationship and its potential influence on risk for future neurodegenerative diseases.^68^ Sex‐specific vulnerabilities or neuroprotective factors are not static across the lifespan and the potential interaction between age and sex on post‐injury outcomes is critical to understand in future research studies. Furthermore, no studies were identified that looked at links between estrogen and TBI during pregnancy. This is particularly important for future research given that pregnancy is a high‐risk period for experiencing a TBI owing to increased incidence and severity of intimate partner violence.^58^

When considering TBI, it is important to note that, while it is likely that the incidence of women experiencing TBI is underreported and underestimated, epidemiological studies consistently report higher incidence in men.^69^ Evidence from the CRASH (Corticosteroid Randomization after Severe Head Injury) dataset reported that older women experienced the worst outcomes of TBI; however, this finding was not replicated in a primary data collection in Bangladesh conducted alongside this analysis.^70^ Looking at a younger population, the TRACK‐TBI team reported a significant interaction between age and sex on outcomes after a mild TBI, whereby females aged 30–39 years had increased symptomology at 6 months compared to younger females and males.^71^ A further article from the Transforming Research and Clinical Knowledge in TBI (TRACK‐TBI) study considering a broader age range reported more persistent cognitive and somatic symptoms after a mild TBI in female compared to male patients, with a significant interaction with age in which the worst somatic symptoms were experienced by females aged 35 to 49 years.^72^

Despite the suggestions around the neuroprotective effect of estrogen, there is evidence of a sex disparity in experience of concussion, with women reported in several studies to have greater vulnerability to concussion and its consequences, including more severe post‐concussive symptoms, than men.^67^ There is some evidence of sex‐related differences in sport‐related concussions, although there are a number of studies finding no differences in sex.^73^ Female veterans may also experience more long‐term post‐concussive sequalae compared to male veterans, despite being less likely to sustain a TBI.^74^

### 3.3 Hypertension

Estrogen has cardioprotective effects by initiating vasodilation to promote blood flow and inhibit atherosclerosis. Menopausal estrogen decline removes these cardioprotective effects.^75^ In rodents, estrogen also modulates a multitude of systems in the body that are implicated in hypertension, including the renin–angiotensin–aldosterone system, sympathetic nervous system, and circadian rhythms.^76^ Nevertheless, most studies report no significant associations between hypertension and age at natural menopause.^77^, ^78^, ^79^

Women who experience hypertension during pregnancy (such as gestational or chronic hypertension, pre‐eclampsia, eclampsia) were shown to be at a higher risk of cognitive decline in later life.^80^ During pregnancy, the placenta synthesizes large quantities of estrogen (mainly estradiol).^81^ During pregnancy, blood pressure typically decreases during the first and second trimesters in parallel with rising estrogen levels.^82^ However, low levels of estradiol, indicative of issues with placental estrogen synthesis, are a major contributor to risk for preeclampsia, occurring in 3% to 7% of all pregnancies.^81^ Estrone and estriol are also reduced in pre‐eclampsia, although this finding is less consistent across studies.^81^

Considering exogenous estrogen exposure, there are several studies that have examined either contraceptives or HRT use and hypertension. Oral hormonal contraceptives have long been known to increase risk for hypertension, with risk returning to that of non‐users rapidly on cessation of oral contraceptives.^83^, ^84^ Other forms of hormonal contraceptives have also been investigated more recently, with a recent systematic review reporting that injectable hormonal contraceptive use, the hormonal intra‐uterine device, and the vaginal ring are all associated with reduced blood pressure across age groups and populations.^85^ Understanding these unintended consequences is helpful for developing a protective profile against future dementia risk. Use of HRT (both estrogen only and combination therapy) is associated with reduced risk of hypertension in postmenopausal women in several studies,^86^, ^87^, ^88^, ^89^, ^90^, ^91^, ^92^, ^93^ although other studies report no significant associations.^94^, ^95^, ^96^, ^97^, ^98^ More research is needed to understand the population strata who may benefit most from cardiovascular benefits of HRT.

Rodent models strongly support estrogen's role in blood pressure regulation. However, more research is needed to understand how changing estrogen levels across the human life course influence hypertension. There is a growing, albeit inconsistent, evidence base demonstrating associations between exogenous estrogen use and changes in blood pressure.

### 3.4 Alcohol use

Gonadal hormones influence alcohol intake and drinking patterns,^99^ with species‐specific sex differences in alcohol sensitivity emerging after late puberty.^100^ While past research has found that men consume more alcohol than women, this sex gap appears to be narrowing at least in data predominantly from North American and European studies.^101^ This finding warrants further attention, as women are more vulnerable to a range of alcohol‐related health concerns such as liver problems, breast cancer, hypertension, and an increased likelihood of psychiatric issues.^102^, ^103^, ^104^, ^105^

Studies have reported associations between higher estrogen and increased alcohol use for females, with mixed evidence for males.^106^ Menstrual cycle phases seem to influence alcohol consumption,^107^ although findings are mixed.^108^ Some studies have found a positive association between alcohol use and luteal estrogen.^109^, ^110^ However, other studies reported a reduction in binge‐ drinking during the late luteal phase,^111^ or higher mean estradiol levels after sustained alcohol intake throughout the entire menstrual cycle.^112^ Yet, research has also demonstrated that drinking and binge drinking may be highest in the late follicular phase^113^ and during ovulation,^114^ when estrogen levels are highest. Other factors may also influence women's alcohol intake during this time, such as social gatherings^115^—or negative emotional experiences.^99^, ^116^ Differences in study populations and covariates included in the statistical models, as well as challenges with recording of alcohol intake, are all likely to contribute to these inconsistent findings. Few studies have investigated links between alcohol use and estrogen during pregnancy when alcohol consumption is discouraged for fetal health. One study from 1992 reported no significant associations between alcohol use in the 26th week of pregnancy and estradiol or estriol (but higher total estrogens), but with higher estriol in the 31st week of pregnancy (no association with estradiol and total estrogens).

Some studies have found that positive associations between alcohol use and estrogen in women may depend on age. One study comparing postmenopausal women found positive correlations between estrogen (both estradiol and estrone), regardless of HRT use.^117^ A recent meta‐analysis confirmed this positive relationship in postmenopausal women, but not premenopausal women.^118^ Postmenopausal women also seem to be at greater risk of alcohol use disorder and mortality than other groups.^119^ Menopause transition is also associated with changes in drinking patterns, with an increased likelihood of engaging in excessive drinking, potentially associated with broader factors such as retirement and widowhood.^120^

While there is a growing body of research investigating alcohol use among women, further research is warranted to clarify its relationship with estrogen across the life course.

### 3.5 Obesity

An analysis of data from > 30,000 participants from the Korean National Health and Nutrition Examination Survey (2010–2020) identified a reverse J shape pattern, whereby both early menarche (before 11 years of age) and later menarche (after the age of 16) were associated with increased body mass index (BMI) and abdominal obesity later in life, as well as a range of other cardiometabolic conditions.^121^ Women with obesity were at a higher risk for menstrual irregularity in a separate analysis of this dataset.^34^ Obesity during pregnancy is linked to worse outcomes, including pregnancy loss, preterm delivery, and pre‐eclampsia. In a comparison of obese and non‐obese pregnant women, there were no differences in any trimester in estradiol levels, although there was a trend toward lower estrone levels in the third trimester when the fetus was female. This was despite significant differences in other sex steroids such as lower testosterone in the first trimester in obese women.^122^

Decreases in estrogen production during menopause have been associated with abdominal fat redistribution, with early menopause linked to weight gain and increased abdominal adiposity.^123^, ^124^, ^125^ Most studies report no significant associations between obesity and age at natural menopause. However, obesity indicators, such as BMI and waist‐to‐hip ratio, showed significant mediating effects on the association between a later age at menopause and higher blood pressure.^126^ There is a lack of robust evidence investigating these associations, and further research is needed into this topic.^127^, ^128^, ^129^

Despite commonly held views among clinicians and patients, there is no empirical evidence linking oral contraceptive use and weight gain.^130^, ^131^ However, women with obesity who use combined oral contraceptives are thought to have greater cardiovascular risk (such as increased chances of developing venous thromboembolisms) and as such may also be at a higher risk for poorer brain health via these risk factors.^132^ There is some evidence that use of hormonal depot or intrauterine device contraceptive methods are associated with weight gain compared to non‐hormonal intrauterine device (copper coil) use.^133^ HRT use during menopause has been associated with reduced adiposity and improved metabolic parameters, although these effects are relatively small in magnitude.^134^

From the studies included in this review, endogenous estrogen appears to be associated with obesity. However, there is less consistent evidence of any association between exogenous estrogen use and obesity.

### 3.6 Smoking

Exposure to cigarette smoke has been associated with estrogen deficiency such as hypoestrogenism.^135^ Cigarette smoke has a toxic effect on reproductive organs leading to ovotoxicity and decreased estrogen hormone secretion.^135^, ^136^ Smokers have also been found to have approximately three times lower urinary excretion of estrogen than non‐smokers.^137^ Smoking has been found to have a direct effect on the menstrual cycles of females who are heavy smokers, with increasingly irregular menstrual cycles correlating with frequency of cigarette smoking.^138^ Similar to alcohol, there are few recent studies that have investigated associations between smoking and estrogens during pregnancy. Older studies that have reported on this tend to find that smokers during pregnancy have lower estrogen levels than non‐smokers.^139^, ^140^ Further to this, smoking has also been associated with early menopause due to its anti‐estrogenic effects.^141^

Smokers who use oral contraceptives may metabolize nicotine faster than non‐oral contraceptive users, with findings specific to estrogen‐containing contraceptives.^142^ This is significant as faster nicotine metabolism is associated with a greater risk of nicotine addiction and more severe withdrawal symptoms, particularly in the first 24 hours without exposure to cigarettes.^143^ Relatively few studies have attempted to understand estrogen‐containing contraceptive use and smoking outcomes, with more research needed on this topic. HRT when taken orally has been found to be ineffective for female smokers, but treatment is effective if given via a transdermal route.^144^

The relationship between smoking and its impact on estrogen production is complex and not fully understood yet. However, it is clear that smoking does impact estrogen levels, which may have wider health implications for women.

### 3.7 Depression

Previous research has found that women are disproportionately affected by depression compared to men.^145^ Women experience a higher risk of depression during times of ovarian hormone change such as puberty, menstruation, postpartum, and menopause.^146^ Estrogen fluctuations may in part explain this apparent sex discrepancy in risk for depression.^147^ Estradiol modulates monoamines such as serotonin, a key neurotransmitter implicated in depression.^148^ Estradiol also influences the activity of emotion‐regulating brain structures; as a result, women may experience changes in their responses to negative emotional stimuli and stressful life events.^147^ Contrastingly, male estrogen research has found that depressive symptomatology in younger men was significantly associated with higher levels of estrogen,^149^ but the increasing depression prevalence in older males does not appear to be driven by sex hormones.^150^

Pregnancy is a known risk period for depressive symptoms, both in the peri‐ and postnatal phases.^151^, ^152^ One proposed mechanism for postnatal depression is the significant decline in estrogen after birth.^153^ However, alternative hypotheses involve progesterone, and there remains a need to investigate whether perinatal depression is linked to estrogen, given it occurs at a time of increasing estrogen levels through each trimester.

Research on the relationship between depression and menopause has found the global prevalence of depression is 35.6% in menopausal women, 33.9% in perimenopausal women, and 34.9% in postmenopausal women.^154^ However, it is not clear if this is directly attributable to reproductive status, or if psychosocial factors present during menopause—such as children leaving home, changes with work, or caregiving for aging family members—also impact depression risk.^155^ Some evidence suggests that vasomotor symptoms of menopause are associated with depressive symptoms, although not with major depressive disorder.^156^

Women who experience earlier menopause have an increased risk for later‐life depression,^157^ and a longer lifetime estrogen exposure, measured as the time interval from menarche to the onset of menopause, seems to be protective against depressive symptoms.^148^ Clinical trials investigating HRT as a treatment for menopausal depression have been promising; however, timing of the intervention appears to be critical for the effectiveness.^158^ Findings indicate that perimenopausal depression can be effectively treated with estrogen alone, or with estrogen combined with antidepressants, although the latter regimen is more effective.^159^ Changes in hippocampal volume related to exogenous estradiol may relate to trajectories of anhedonia, with further research needed to understand this mechanistic pathway.^160^ However, these apparent anti‐depressant qualities of HRT may be lost in postmenopausal women,^161^, ^162^ suggesting that earlier HRT treatment is more beneficial for menopausal women suffering from depression. Alongside this, age of treatment must also be considered, with evidence that HRT use prior to age 50 associated with an increased risk of depression while treatment after the age of 54 was associated with a decreased risk of depression.^163^

There are well‐established links between estrogen and depression across a woman's life course. However, further research is needed to understand the impact of broader socioeconomic and psychosocial factors on this relationship. HRT may offer some benefits on depressive symptoms when used early in the menopausal transition.

### 3.8 Physical inactivity

Research on the relationship between women's physical activity and estrogen levels often focuses on different body parts or on different aspects of health, such as skeletal deterioration and bone health, obesity, body composition/weight, and muscle mass and strength.^164^, ^165^, ^166^, ^167^ During pregnancy, women may gain muscle strength although this is not associated with changes in estrogen levels. There is a lack of studies investigating hormonal changes associated with physical (in)activity during pregnancy.^168^ While physical activity across the life course is beneficial to women's health,^169^ women tend to become more physically inactive as they enter menopause^170^ and their estrogen levels begin to decline. Indeed, observational studies have found an inverse association between estrogen and physical activity in postmenopausal women.^171^ In contrast, a study on adolescent girls found that urine concentrations of estrogen and other hormones were not influenced by physical activity,^172^ indicating differences between females pre‐ and post menopause. The decline in estrogen for menopausal women is believed to impact many aspects of health, with menopausal women being more at risk of developing multiple different conditions such as osteoporosis,^173^ sarcopenia,^165^ frailty,^174^ and reduction of muscle function.^164^ These changes may contribute to the development of diabetes or obesity,^166^ which in turn can severely impact menopausal women's physical activity, or lead to a lack thereof.

Some studies found that HRT can counteract some of the negative physical changes associated with menopause.^175^ HRT has been shown to increase muscle strength in postmenopausal women,^167^ may reduce the risk of lower limb ligament and tendon injuries,^164^ and increase bone mineral density (BMD).^176^ One randomized clinical trial found no association between HRT and physical activity levels in postmenopausal women.^177^This contrasts with findings from rodent studies suggesting that greater female (compared to male) physical activity is mediated by estrogenic mechanisms (see Lightfoot^178^ for a review).

Little research has investigated links between estrogen and engagement in physical activity, limiting the ability to draw any conclusions on associations with this potentially modifiable risk factor for ADRD.

### 3.9 Diabetes

Increasing evidence suggests that diabetes may result from an imbalance of sex hormones in both men and women.^179^ Estrogen appears to be protective for female metabolic health due to its influence on the distribution of body fat mass, mobilization of fatty acids, and the glucose response from tissues and organs.^180^ Type 1 diabetic adolescents exhibit lower serum estrogenic activity and estradiol levels than controls.^181^ However, research is currently inconclusive regarding whether estradiol is implicated in type 2 diabetes as studies report conflicting results.^179^ Some studies have found associations between elevated estradiol and diabetes incidence, whereas others have found no association.^182^

A younger age at menarche is associated with a higher risk for pre‐diabetes and diabetes. This relationship may be partially explained by BMI, adiposity, childhood socioeconomic status, and genetic factors,^183^, ^184^, ^185^, ^186^ although these findings are not consistently replicated in all analyses,^187^ with differences seen depending on age of cohort studied.

Women with gestational diabetes during pregnancy have been found to exhibit higher levels of estriol, estrone, and estradiol compared to women without gestational diabetes, with first trimester estrone levels showing a particular association with later development of the condition.^188^, ^189^

Metabolic changes, including insulin resistance, increase during the menopause transition when estrogen levels decline, and estrogen HRT has been shown to reduce this risk.^180^ Surgically induced menopause has also been associated with an increased risk of adverse metabolic changes and insulin resistance.^190^ HRT use appears to reduce the incidence of type 2 diabetes in menopausal women; however, the outcomes differ dependent on the route of administration and type of treatment used.^191^

More research is needed to understand what, if any, associations exist between estrogen and diabetes, and whether HRT can effectively moderate this risk.

### 3.10 High low‐density lipoprotein cholesterol

There is substantial evidence linking estrogen to low‐density lipoprotein (LDL) cholesterol, both in terms of endogenous and exogenous estrogen exposure. Estrogen is created through steroidogenesis from LDL cholesterol.^192^, ^193^ Estradiol levels negatively correlate with LDL cholesterol across the life course. Moreover, LDL cholesterol levels fluctuate throughout the menstrual cycle, whereby levels are lowest in the ovulation phase, higher in the luteal phase, and highest in the follicular phase.^194^ LDL cholesterol levels also rise throughout pregnancy, in parallel with rising estrogen levels.^195^ The menopausal transition appears to herald increased risk for dyslipidemia,^196^, ^197^, ^198^ and HRT use has been shown to improve lipid profiles in multiple studies,^198^, ^199^ although HRT use did not contribute to conversion to dementia in subgroups of women with extreme ranges of LDL cholesterol levels.^200^ Surgical menopause may further increase the risk for metabolic syndrome, which includes changes in LDL cholesterol.^196^ This may relate to the earlier average age of menopause and increased prevalence and severity of menopause symptoms in those undergoing surgical menopause.^201^

Use of hormonal contraceptives is associated with limited changes to lipid metabolism, with newer generation formulations (containing estradiol) posing fewer metabolic effects compared to older formulations.^202^

## 4 ASSOCIATIONS BETWEEN LATER‐LIFE MODIFIABLE RISK FACTORS FOR DEMENTIA AND ESTROGEN

### 4.1 Social isolation

Few studies have investigated associations among estrogen, hormonal contraceptive use, or HRT and social isolation. As social isolation is largely driven by social issues and social constructs rather than biological mechanisms, research exploring sex and gender interactions with social isolation may provide more insights than studies focused solely on associations with estrogen, particularly when aiming to identify sex‐ or gender‐specific risks or protective factors for social isolation. For example, a recent study of > 12,000 community‐dwelling older adults in Australia found that persistent loneliness was associated with a higher risk of dementia, particularly in women, but social isolation was associated with cognitive decline but not risk for dementia.^203^

### 4.2 Air pollution

Exposure to air pollution such as nitrogen dioxide (NO_2_), ozone (O_3_), and particulate matter (PM)—especially fine PM that is ≤ 2.5 microns in diameter (PM_2.5_)—has been associated with an increased risk of dementia.^204^, ^205^, ^206^ Air pollutants can impact the brain through neuroinflammation and neurodegeneration mechanisms either directly via the olfactory pathway and crossing the blood–brain barrier (BBB), or indirectly via systemic inflammation through the body and oxidative stress, which can further disrupt the BBB integrity.^207^, ^208^, ^209^

Certain air pollutants can be considered endocrine disruptors, potentially interfering with hormone production and activity, including estrogen.^210^, ^211^ Increased NO_2_ concentration during early pregnancy (gestational weeks 0–15) has been associated with higher estrone and estradiol concentrations; however, later NO_2_ concentration increase (gestational weeks 22–30) was associated with lower estradiol concentrations, suggesting that timing of air pollution exposure is important for estrogen during pregnancy.^212^

A longitudinal United States–based multi‐ethnic study found that exposure to air pollution (PM_2.5_, NO_2_, and O_3_) during the menopausal transition was associated with significant declines in sex hormones, such as estradiol and follicle‐stimulating hormone, potentially exacerbating menopausal symptoms and associated health risks.^213^ A greater exposure to environmental pollution may also lead to accelerated ovarian aging mechanisms and earlier menopause.^214^ In contrast, a cross‐sectional study in China found that PM_2.5_ exposure, composed of both water‐soluble and inorganic elements, was associated with increased estradiol levels.^215^ Ambient PM collected in Italy, particularly in urban areas and during winter, exhibited significant estrogenic activity in a gene reporter assay. This activity correlated with the concentration of polycyclic aromatic hydrocarbons (PAHs), suggesting the endocrine system–disrupting potential of PM interfering with the functioning of estrogen.^216^ A German cohort study found a genetic polymorphism in the estrogen receptor beta (ESR2), which can impact the function of estrogen signaling, was associated with cognitive function, and ESR2 alleles interacted with enhancing the relationship between air pollutants (PM_2.5_ and NO_2_) and reduced cognitive function.^217^ Women are more sensitive to the effects of air pollution before age 15 and after age 65, which may suggest that reduced exposure to sex hormones could influence women's susceptibility to air pollution.^218^ Research in rats has found that estrogen treatment is protective against neurodegeneration and oxidative stress from ozone inhalation.^219^ These findings suggest that age‐related reductions in hormones may be related to sex‐specific susceptibility of air pollution effects.^208^

### 4.3 Untreated vision loss

Myopia is an increasingly common diagnosis and has been reported as more common in females compared to males.^220^ A study of adolescent females (aged 15 and 16 years, *n* = 120) found significant changes in lens thickness and visual acuity throughout the menstrual cycle, varying in relation to circulating estradiol levels.^221^

Oral contraceptive pill use has been associated with deep microvascular changes in the retina as measured using optical coherence tomography (OCT), suggesting that vascular changes associated with oral contraceptives in other organs may also affect the eye, and may have risks for future retinal vascular disease that could warrant monitoring in higher risk individuals.^222^ Similarly, oral contraceptive pill use was associated with lower macular, retinal nerve fiber layer, ganglion cell layer, and choroidal layer thickness measured using OCT.^223^, ^224^

One year of HRT has been associated with improved visual function, better contrast sensitivity, and higher tear production, but not intraocular pressure, compared to a control group in a small (*n* = 80) randomized control trial.^225^ Longer duration of HRT (≥ 10 years) was associated with a reduced risk of nuclear and posterior subcapsular (but not cortical) lens opacities in the Framingham Health Study, suggesting a benefit for visual health from postmenopausal HRT use.^226^ Although glaucoma was not linked to dementia risk in one of the contributing meta‐analysis studies considered by the Lancet Commission,^33^, ^227^ there is a noted higher burden of glaucoma in older women, with evidence to suggest that loss of estrogen through earlier menopause is associated with increased risks for glaucoma and wider optical damage caused by this condition.^228^

## 5 ASSOCIATIONS BETWEEN NON‐MODIFIABLE RISK FACTORS FOR DEMENTIA AND ESTROGEN

### 5.1 *APOE* ε4

A study of young, regularly menstruating women in Poland reported no differences in estradiol levels in *APOE* ε4 carrier status; however, the study did report that *APOE* ε4 carriers had significantly higher luteal progesterone compared to non‐carriers.^229^

Limited studies have investigated associations between *APOE* ε4 carrier status and hormonal contraceptive use. One study investigated the potential interactive effects between *APOE* ε4 status and hormonal contraceptive use on cognition in midlife women (mean age 52.4 years), with no significant interactions identified.^230^ Positive associations between HRT use, allocentric processing, and hippocampal volume were only observed in *APOE* ε4 carriers and not in *APOE* ε4 non‐carriers (mean age 65.1 years) in the European Prevention of Alzheimer's Dementia Longitudinal Cohort Study.^26^ Similarly, a study of 300 women (never users mean age 72.5 years, past users mean age 71.9 years, current users mean age 70.6 years) found that while cognitive performance declined over 6 years in all non‐users and current HRT users who were not *APOE* ε4 carriers, *APOE* ε4 carriers and HRT users did not show cognitive decline during the study period.^231^ Conversely, a study of 134 cognitively unimpaired women (mean age 65.95 years) enrolled in the Wisconsin Registry for Alzheimer's Prevention (WRAP) cohort study reported that HRT users who were *APOE* ε4 carriers (*n* = 27) had higher AD biomarker concentrations (phosphorylated tau [p‐tau]/amyloid beta [Aβ]42 ratio and Aβ42/Aβ40 ratio) compared to non–*APOE* ε4 carriers (*n* = 53).^232^

A recent prospective study investigated the effects of 6 months of estrogen therapy in HRT‐naive, cognitively healthy menopausal women (mean age 54.03 years), finding longitudinal changes in p‐tau/Aβ40 ratios among women in the HRT group, with the most pronounced effects observed in *APOE* ε4 carriers.^27^

## 6 ASSOCIATIONS BETWEEN POTENTIAL MODIFIABLE RISK FACTORS FOR DEMENTIA AND ESTROGEN

### 6.1 Sleep

There are well‐documented differences in how men and women sleep. For example, women postmenarche are more likely than men to experience insomnia.^233^ Women also tend to report needing more sleep than men in multiple studies.^233^ Although some studies have suggested that there are differences in how deeply women sleep compared to men, a recent systematic review found no high‐quality evidence for sex differences in alpha and delta power on electroencephalogram measures in midlife.^234^

Evidence from a systematic review found that higher endogenous estrogen was associated with better sleep in women without depression.^235^ The review also considered the interplay among depression, estrogen, and sleep but did not find conclusive evidence. The luteal phase of the menstrual cycle appears to correlate with poorer sleep, particularly for those with high luteal progesterone.^235^ Sleep quality and quantity is affected throughout pregnancy and into the fourth trimester. In a review of the topic, authors summarize that sleep disruption (both subjectively and objectively measured) is common from the first trimester through to the immediate postpartum period.^233^ Although there are significant changes to hormones throughout this time, studies have not been able to disentangle other physical changes that may drive these sleep changes, such as increased urinary frequency and physical discomfort.^233^

Sleep disturbance is a major recognized symptom of menopause, with up to 60% of women complaining of changes to their sleep during this period.^236^ Sleep appears to worsen in menopause beyond what would be expected from normal changes with age, although significant evidence is from subjective reports of sleep and there remains a lack of objective sleep studies during the menopausal transition.^237^ The most common changes appear to be increases in nighttime awakenings and spending more time awake during the night. Significantly more research is needed to understand the etiology of sleep changes during the menopausal transition; however, it is likely a combination of hormonal changes as well as vasomotor symptoms, that drive this.^238^

There is some evidence that suggests an association between hormonal contraceptive use and symptoms of insomnia.^235^ However, a robust series of meta‐analyses of subjective and objective (polysomnography) measures found only one statistically significant difference, with contraceptive users having a slightly (≈ 7 minutes) shorter wake after sleep onset compared to non‐contraceptive users.^239^ However, the analyses were limited by a lack of consistency in measurement of sleep, meaning few studies contribute to each analysis, and type of hormonal contraceptive used. More research would be valuable to understand what, if any, effects there are of hormonal contraceptive use on sleep. Considering HRT, a meta‐analysis including data from 42 trials reported that HRT use had a modest effect on improved sleep quality in women who experienced both sleep problems and vasomotor symptoms, but this effect was not seen in women without vasomotor symptoms.^240^ The evidence base does remain mixed, with studies looking at subjective measures of sleep tending to report positive associations, and less consistent evidence when objective measures are used.^241^

In summary, there is evidence suggesting sex differences in sleep between men and women; however, more research is needed to understand what role, if any, endogenous and exogenous estrogen has in the underlying mechanisms of these differences.

### 6.2 Diet

Much of the evidence around associations between diet and estrogens come from the field of breast cancer research, exploring whether dietary choices or interventions can modify breast cancer incidence.^242^ The Mediterranean diet is the predominant dietary pattern that emerges as best for preventative efforts against breast cancer,^242^ with the same found in reviews of diet and dementia.^243^ However, more evidence is needed to understand associations between specific nutritional components and estrogen concentrations.

Energy intake needs fluctuate across the menstrual cycle, which has an impact on dietary requirements. For example, energy needs are lower in the follicular phase compared to the luteal phase, with increases reported in studies ranging from an additional 90 kilocalories (kcal) a day up to > 500 additional kcal a day.^244^, ^245^ Dietary patterns before and during pregnancy that reflect the Mediterranean style of eating have been associated with a reduced risk of pre‐eclampsia and gestational hypertension in healthy White women, with some evidence to suggest reduced risk of gestational diabetes.^246^ Limited evidence exists for non‐White women or those with pre‐existing health conditions. No studies were identified that look at associations between diet and estrogens during pregnancy. Dietary intake has been associated with earlier age of menopause onset, such as intake of green and leafy vegetables, while high consumption of dairy products has been associated with later onset of menopause; however, it is important to note that evidence is from observational studies with robust high‐quality evidence and replication studies missing.^247^ A small study of 115 postmenopausal women (58 intervention, 57 control) evaluated the effects of the Mediterranean diet on estrogen. After 6 months, women in the dietary intervention group had a significant decrease in urinary estrogens compared to the control group, suggesting that the Mediterranean diet (or components of it) may be able to reduce estrogen levels in postmenopausal women.^248^ While this may be a beneficial preventative measure of breast cancer, research needs to consider implications of this for dementia and explore how these findings might fit in the broader evidence of associations between the Mediterranean diet and reduced risk for dementia. No studies were identified that specifically looked at the interplay between diet and exogenous estrogen in the form of either hormonal contraception or HRT.

The phytoestrogen isoflavone seems to impact the menstrual cycle and hormone levels of premenopausal women, with one study finding an extension in premenopausal women's follicular phase after consuming soybeans with 45 mg of isoflavones.^249^ Consumption of soybean isoflavones can also be shown to decrease the blood follicle stimulating hormone and luteinizing hormone in premenopausal women. The review does note that effects of soy‐protein consumption on hormone consumption is conflicting due to variances in methodology (isoflavone type and dosage, length of the diet, etc.). A recent review highlights sex differences in the hormonal effects of dietary phytoestrogens. In postmenopausal women, studies have shown positive outcomes, including lower LDL cholesterol and better insulin sensitivity, thought to arise from the estrogen‐like effects of one type of phytoestrogen (isoflavones). These plant compounds may help partially replace the role of natural estrogen, leading to improvements in metabolic and cardiovascular health. The review also notes that evidence on phytoestrogens from other reproductive stages (i.e., pregnancy and childbirth) is limited and inconsistent. There is also inconsistent evidence highlighted in a review of phytoestrogen effects on postmenopausal health concerns, including vasomotor symptoms, cancer, cognition, cardiovascular disease (,), and bone health.^250^

Consistent and high‐quality evidence for the impact of diet and estrogen across the life course is lacking. There is a significant need to understand more about these associations.

### 6.3 Infection

Males (both human and other species) appear to be more susceptible to infections than females are,^251^ with differences in susceptibility to, and sequelae of, bacterial and viral infections, as well as differences in the immune response.^252^, ^253^, ^254^ It is likely that these differences are driven by a complex picture of sex steroids as well as genetic and epigenetic factors. Estrogens are known to interact with, and regulate, both inflammation and immune pathways, altering the responses of host cells to microbes. In viral and bacterial infection and pathogenesis, estrogens and estrogen receptors regulate innate immune responses, such as by preventing the production of the type 1 interferons which are important in the immune cascade, and by suppressing proinflammatory cytokines.^255^

Some viruses are known to change the duration or regularity of the menstrual cycle, such as human immunodeficiency virus (HIV), hepatitis B, hepatitis C, and severe acute respiratory syndrome coronavirus 2 (SARS‐CoV‐2), although studies on this topic do not report on underlying associations with estrogen levels in relation to these changes.^256^ Pregnancy is a unique period for the immune system, with adaptations to protect the mother and the developing fetus, reducing risk of immune‐mediated fetal rejection while providing a mechanism to transfer maternal antibodies to the developing fetus.^257^ During this time of complex physiological and immune changes, viral infections can be associated with significant adverse effects for both the mother (such as SARS‐CoV‐2) and the fetus (such as the rubella virus). Estrogens are important for stimulating placental vascular endothelial growth factor, stimulating the secretion of proinflammatory cytokines (particularly in the first trimester), and supporting an environment in the second and third trimesters in which there are reduced proinflammatory and increased anti‐inflammatory cytokine concentrations^258^ (see Creisher and Klein for a detailed reviewed of the pathogenesis of viral infections during pregnancy^258^). Urinary tract infections (UTIs) are one of the most common infections seen within the health‐care system, with both pregnant and postmenopausal women at a high risk of non‐recurrent and recurrent UTIs.^259^, ^260^ Recurrent UTIs can have many serious complications, ranging from sepsis to increased risk for, or acceleration of, several neuropsychiatric disorders.^259^, ^261^ Changes in estrogen concentrations during the menopausal transition are thought to be one of the explanatory factors behind this increased risk for recurrent UTIs.^259^ Exogenous estrogen, in the form of local vaginal therapies, is a recommended treatment option for the prevention of UTIs in this population, although the evidence for the use of systemic estrogen is weaker.^259^ Further research is needed to uncover associations between estrogens and infections in the context of hormonal contraceptive and HRT use.

Sex differences in susceptibility to infections and immune responses are well established, with some evidence suggesting that estrogen is one of the key drivers of these differences. Understanding how estrogen levels interplay with genetic factors in relation to infection, and implications for future dementia risk of any interactions, will be important for future research.

### 6.4 Stress

Post‐traumatic stress disorder (PTSD) is briefly considered in the Lancet Commission report; however, the evidence is insufficient at this stage to draw firm conclusions on overall risk for dementia.^33^ However, it may be an important risk factor to consider through a sex and gender lens, with the prevalence of PTSD among women twice that of men,^262^ although this disparity tends to minimize later in life.^263^ More broadly, there is an evidence base suggesting a role of stress and the hypothalamic–pituitary–adrenal (HPA) axis in the occurrence and progression of neurodegenerative diseases and cognitive disorders.^264^, ^265^ There are well‐established differences in the function of the HPA axis by sex, from development through puberty and during stress reactions.^266^, ^267^ Broader than the scope of this review, there are also a number of important sex differences in stress response that are pertinent to consider in any learnings from the below synthesis of evidence through the lens of sex.^268^

There is a small body of evidence suggesting PTSD‐related flashbacks may be more frequent during the luteal phase, and women who experience a traumatic event during their luteal phase of the menstrual cycle may in turn experience more flashbacks.^269^ However, other findings align more with studies of anxiety, finding increased PTSD symptomology in the premenstrual phase.^269^ Beyond this, some evidence suggests an association between estradiol levels and deficits in extinction learning in PTSD, which may account for worsening of symptoms seen in some studies during the luteal phase when estradiol levels are higher.^269^ Considering associations more broadly between the menstrual cycle and HPA axis functioning, a recent meta‐analysis found significantly higher cortisol levels in the follicular compared to the luteal phase when combining data from 778 participants across 35 studies.^270^ The authors of this meta‐analysis suggest a mechanistic role of estradiol in the regulation of corticosteroid‐binding globulin (CBG) within the broader mechanistic underpinnings of this association. A second meta‐analysis looking at HPA axis reactivity, rather than activity, found conversely that cortisol reactivity is higher in the luteal than follicular phase, although this was only evidence from the analysis of three studies with sufficient data.^271^

Increasing evidence indicates that oxidative stress can impair estrogen signaling pathways, and in doing so can impact uteroplacental circulation. Normal pregnancy is already believed to be a state of oxidative stress,^272^ but a heightened oxidative stress is associated with pregnancy complications such as preeclampsia and IUGR (intrauterine growth restriction), with recent research consistently showing lower serum estrogen levels in women diagnosed with pre‐eclampsia compared to controls.^273^ While the association between high oxidative stress and pregnancy complications is known, the mechanisms behind this relationship are not well understood as the pathogenesis of such complications are under‐researched.^274^

PTSD or experience of trauma were associated with earlier surgical menopause in the Nurses’ Health Study II, but not with earlier age at natural menopause, with no data presented about estrogen levels during this period.^275^ A cross‐sectional study using an urban community sample from a public hospital in Atlanta, USA, reported higher perimenopausal symptoms in women who had higher PTSD symptom severity, compared to pre‐ and postmenopausal women, although there were again no data on estrogen levels to understand any associations among this, PTSD, and the experience of menopausal symptoms.^276^

A small study (*n* = 33 women, 20 using hormonal contraception, 13 not using hormonal contraception) reported that women using hormonal contraceptives had enhanced fear conditioning and extinction of fear compared to women not using hormonal contraceptives and men (*n* = 48).^277^ Results from a meta‐analysis including data from 1279 participants across 14 studies found significant blunting on the HPA axis for oral contraceptive users compared to naturally cycling females after exposure to the Trier Social Stress Test, suggesting further research is needed to understand implications for conditions such as PTSD, including the risk for, and management of symptoms.^278^ Using data from the Atlantic Partnerships for Tomorrow's Health (PATH) study, there was no significant difference in PTSD diagnosis between postmenopausal women who had never used HRT or those on estrogen‐only or estrogen‐progesterone combined HRT; however, PTSD diagnosis rates were higher among progesterone‐only HRT users.^263^ A small study of 14 HRT users and 14 non‐users (plus 14 younger premenopausal participants) found HRT users had morning salivary cortisol levels comparable to younger women, and lower than non‐HRT users, suggesting a possible role of HRT in attenuated age‐related changes in HPA axis functioning.^279^

Although there are well‐established sex differences in PTSD and HPA axis functioning by sex, there is more limited evidence of association with endogenous and exogenous estrogen across the life course with these stress conditions. As research continues to uncover the implications of PTSD and HPA axis functioning for neurodegenerative disease initiation and progression, understanding this through the lens of sex or gender will be important for identifying underlying mechanisms and potential intervention opportunities.

## 7 DISCUSSION

**FIGURE 1:**
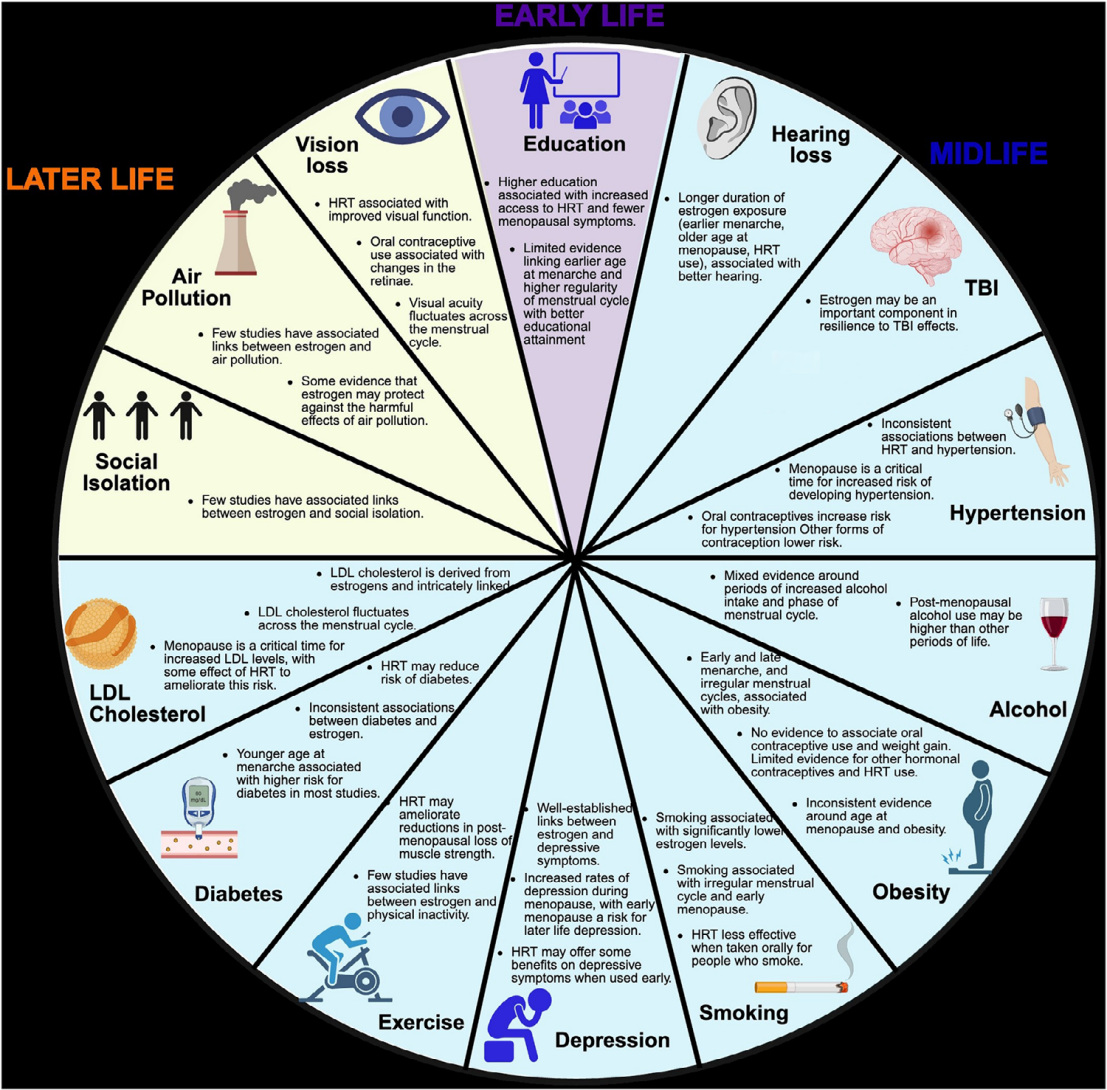
Overview of reported associations in the synthesized literature between estrogen and 14 modifiable risk factors for dementia (early life: education; midlife: hearing loss, TBI, hypertension, alcohol, obesity, smoking, depression, exercise (physical activity), diabetes, LDL cholesterol; later life: social isolation, air pollution, vision loss. HRT, hormone replacement therapy; LDL, low‐density lipoprotein; TBI, traumatic brain injury.

In this review, we synthesize evidence on associations between estrogen—specifically age at menarche and menopause, pregnancy, menstrual cycle patterns, and exogenous estrogen use (contraception, HRT)—and known potentially modifiable risk factors for dementia, *APOE* ε4, as highlighted by the 2024 Lancet Commission on dementia prevention, intervention, and care.^33^ An overview of the findings for the 14 potentially modifiable risk factors is presented in Figure 1. For some risk factors, such as LDL cholesterol, smoking, and depression, there is a growing body of research to draw upon; however, for other areas, such as air pollution and alcohol and the emerging risk factors for dementia, there is limited evidence.

The evidence for association between LDL cholesterol and estrogen is notable, given the steroidogenic pathway that converts LDL cholesterol into estrogen.^192^, ^193^ This fundamental association between the two compounds is reflected through the life course, with estradiol levels inversely associated with LDL cholesterol, clear fluctuations in concentrations of both throughout the menstrual cycle, and significant changes in both possible at the time of menopause. A recent systematic review and meta‐analysis, synthesizing evidence from 73 studies, found that HRT use significantly reduced LDL cholesterol levels compared to placebo or no treatment, with estrogen‐only treatments having more favorable effects than combined estrogen–progesterone regimens.^280^

Smoking is also strongly associated with estrogen. Chronic smoking is linked to significantly lower estrogen levels and is associated with menstrual irregularities, earlier menopause, and reduced HRT efficacy. Smoking cessation is associated with a return to expected estrogen levels in postmenopausal women, suggesting that changing smoking behaviors may benefit hormone concentrations.^281^ However, smoking cessation may be more difficult for female smokers compared to males, due to differences in responses to nicotine, regulation of stress hormones, and weight management in the postmenopausal period.^138^, ^282^

Depression and depressive symptoms have also been associated with estrogen in several studies. While an important factor to consider across the life course, menopause appears to be a time of critical importance for the emergence of depressive symptoms, with early menopause a particular risk for later life depression.^157^ This is both true for women who have never experienced depression before, and for those with a history of depression who are at an increased risk of relapse during this period of their lives.^283^ Given that depression is both a risk factor for dementia and a feature of the dementia prodromal phase,^33^ minimizing peri‐ and postmenopausal depressive symptoms may be important for reducing an individual's overall risk profile for dementia. While there is some evidence that using HRT early, during perimenopause, is likely to be beneficial, it is not an effective postmenopause treatment, when more effective treatment options are needed.^284^, ^285^

Many other risk factors have a burgeoning evidence base, which suggests that, at least during menopause, there may be benefits from maintaining estrogen levels to reduce the sequalae of rapid estradiol decline. These include maintaining better hearing and vision and reducing risks for hypertension and diabetes. Significantly, more research is needed to better understand associations between estrogen and these risk factors, as well as the remaining risk factors for which the evidence base is currently poor (e.g., education, alcohol use, air pollution).

Cerebrovascular health is increasingly recognized as a pathway linking estrogen to dementia risk. Estrogen plays a role in cerebrovascular health, especially in relation to cerebral small vessel disease (SVD), with evidence showing prolonged exposure to endogenous hormones associated with lower SVD burden in postmenopausal women.^286^ The vascular protection conferred by estrogen is well established and includes accelerated endothelial recovery after cerebrovascular injury,^287^ angiogenesis, enhanced blood flow, protection against oxidative stress, and more.^288^ While females have lower incidence of (naturally occurring) stroke, the loss of estrogen after menopause eliminates this female protective advantage.

This effect extends to cardiovascular risk factors too: while CVD is more prevalent in men under 50 than in premenopausal women, CVD incidence in women increases after menopause, eventually exceeding that of men.^289^

While this review has focused on estrogen, this is likely to be only one contributor accounting for sex differences seen in risk for ADRDs. Sex‐based analyses are essential to uncover many other mechanisms and opportunities for intervention. For example, social isolation is more likely to be socially, rather than biologically, driven. In an Australian study involving community‐dwelling older adults, social isolation was associated with worse cognitive function in women, but not men, despite the men in the study reporting more social isolation.^290^ Studies have demonstrated that women tend to have larger, and more diverse, social connections compared to men, although interestingly an analysis of the same Australian cohort found that women with the largest networks were most at risk of dementia, suggesting that it is not quantity, but quality, that matters.^291^ Furthermore, recent research has highlighted how structural and social inequalities, such as sexism, may contribute to sex differences in cognitive decline. For example, a recent study found that women born in the United States with higher levels of structural sexism (as assessed by levels of unequal labor force participation, political representation, and poverty rates) experience significantly faster declines in memory later in life compared to those born in less sexist states.^292^ This association was found to be particularly strong among Black women, reflecting dual effects of sexism and racism on health outcomes. These findings highlight the importance of considering not only individual‐level risk factors, but also the broader contexts in which women live, as these contexts may influence factors such as access to health care and resources.

Several complex medical, social, and cultural factors must be considered when making recommendations about exogenous estrogen use, both in the form of hormonal contraception and HRT. From a medical perspective, both contraception and HRT are associated with increased risk of venous thromboembolism.^293^, ^294^ In addition, HRT is associated with an increased risk of breast cancer (the risk reported as of most concern to patients and clinicians), stroke, and a detrimental impact on bone and cardiovascular health with long‐term use,^294^ as well as risk of dementia in some studies reported above.

Social and traditional media can also impact attitudes and behaviors regarding the use of hormonal contraception and HRT. Social media campaigns have arisen in recent years advocating for natural alternatives have promoted a move away from hormonal contraception, driven by concerns about side effects and autonomy.^295^ Discussion of menopause care in the media has been associated with increased requests for HRT, with social media content also thought to drive changes in women's decision making around HRT use.^296^ Conversely, negative media coverage about menopause treatments can drive reductions in HRT use or increase concerns about use of these therapies.^297^

In the United States, access to exogenous hormones is shaped by restrictive health‐care policies impacting women, both generally and disproportionally affecting minoritized communities. Access to medical insurance coverage for hormonal contraception or HRT is limited for low‐income and minority populations. Additionally, recent restrictions on abortion services and family planning must also be considered, with implications for the future US research on estrogen and brain health.^298^ Finally, cultural dynamics/differences can influence decision making about use of hormonal contraception or HRT. For example, in many countries, particularly in the Global South, male partners significantly impact decisions about contraceptive use, although this differs depending on whether it is a decision about general use, or also deciding on the type of contraceptive used.^299^ In the Global South there is also commonly a disapproval of premarital sex, which in turn has implications for reduced access to contraception for younger, unmarried women.^299^ In many countries, family members’ views are also part of the decision‐making process about the use of hormonal contraception^299^ and HRT.^297^ These wider factors must be considered both in the context of any recommendations for hormonal contraception or HRT use, and when planning future research studies, as estrogen therapies do not exist purely in the medical domain but have important social, political, and cultural components. The decision ultimately, in regions where there is free access to exogenous estrogen, should always be individualized to the patient. Potential effects on future dementia risk, be they beneficial or detrimental, are an important part of a wider conversation about benefits and risk of the use of hormonal contraception or HRT.

## 8 LIMITATIONS

Several limitations of this review should be noted. Many of the studies included were conducted within the context of cancer research, particularly breast cancer. This focus may limit the generalizability of some of the evidence to a non‐cancer population and highlights the broader need for more women's health research in general, beyond female‐specific disease contexts.

Additionally, this review did not examine the potential interactions between biological and social factors in depth. For example, age of menarche, poorer pregnancy outcomes, and age at menopause have all been associated with lower socioeconomic status.^36^, ^300^, ^301^ Lower socioeconomic status is also associated with increased risk for dementia,^302^ and greater prevalence of modifiable risk factors such as smoking,^303^ obesity.^304^ and alcohol use^305^ on both an individual and community level. These disparities extend beyond socioeconomic dimensions to ethnicity and race, which can shape menopausal timing and transition experiences.^306^, ^307^ attitudes toward health‐seeking behaviors,^308^ and overall dementia risk profiles.^33^

Furthermore, while the Lancet Commission on dementia prevention, intervention, and care provides a comprehensive framework for modifiable risk factors across the lifespan, key biological mechanisms that influence cognitive resilience, particularly the role of neuroprotective steroids such as estrogen which we have focused on, were not fully considered. Estrogen has well‐documented interactions with risk factors not captured within the Lancet Commission report such as sleep, diet/nutrition, and stress regulation, all of which are critical to brain aging and dementia risk.^309^ Given the established neuroprotective effects of estrogen on synaptic plasticity, vascular integrity, and metabolic function,^310^ its decline across the menopause transition represents a unique vulnerability for women, warranting further investigation.

Future studies should prioritize research on the complex interplay among endocrine aging, modifiable lifestyle factors, and cognitive decline, particularly in diverse populations in which socioeconomic and cultural determinants may amplify risk differentials. Many of the studies identified were only cross‐sectional in nature, limiting insights into long‐term health outcomes. A persistent methodological challenge is the accurate measurement of estrogen levels. An alternative is BMD,^311^ as estrogen receptors are expressed in bone tissue, and BMD is positively correlated both with endogenous and exogenous estrogen exposure. BMD has also been shown to be strongly associated with cognitive decline and may serve as a proxy marker for cumulative estrogen exposure over the life course.

## 9 RECOMMENDATIONS FOR FUTURE WORK

Significant research is needed to understand these factors beyond cross‐sectional associations and to investigate the longitudinal implications of estrogen across the life course. Many potentially modifiable risk factors for dementia are co‐morbid, and understanding the complex association between single, and co‐morbid diagnoses and estrogen will be critical to identifying intervention opportunities. Understanding the impact of female‐specific risk factors on brain health in later life will also be important in the move toward precision medicine approaches. Research developed in this field should continue to increase participation from underrepresented communities, to ensure that the findings are applicable to all women and gender‐diverse individuals.

There is also evidence that age of menarche is occurring earlier globally.^312^ Understanding the long‐term consequences of this shift for brain and cognitive health in younger generations of women is an important research priority.

Female sex is associated with higher incidence and risk of ADRD. Estrogen may be a key mechanism contributing to this increased risk in women. Current evidence suggests that estrogen is associated with several modifiable dementia risk factors, particularly LDL cholesterol, smoking, and depression. Further research is required to define these associations and determine whether targeting estrogen levels at specific periods across the life course may offer opportunities to reduce future dementia risk in women.

## CONFLICT OF INTEREST STATEMENT

Sarah Gregory and Miles Welstead are employees of Scottish Brain Sciences; Mariapaola Barbato is the scientific lead of the Women's Brain Foundation. Antonella Santuccione Chadha is the co‐founder and pro bono CEO of the Women's Brain Foundation, and is also the pro bono Euresearch vice president. Craig W. Ritchie is founder and CEO of Scottish Brain Sciences. All other authors have nothing to declare. Author disclosures are available in the supporting information.

## CONSENT STATEMENT

This article does not contain any data from human participants or animals, hence informed consent was not required.

## Supporting information



Supporting Information

## Appendix Search Terms

Modifiable risk factors:

“education,” “hearing loss,” “traumatic brain injury,” “TBI,” “hypertension,” “alcohol,” “obesity,” “BMI,” “smoking,” “tobacco,” “vaping,” “e‐cigarettes,” “depression,” “depressive symptoms,” “loneliness,” “social isolation,” “physical inactivity,” “diabetes,” “air pollution”

Non‐modifiable risk factors:

“APOE,” “APOEe4”

Estrogen‐related factors:

“estrogen,” “estrone,” “estriol,” “estradiol,” “hormonal contraception,” “hormone replacement therapy,” “HRT,” “pregnancy,” “menopause”
